# Point-of-care testing in primary care: needs and attitudes of Irish GPs

**DOI:** 10.3399/bjgpopen17X101229

**Published:** 2017-11-15

**Authors:** Laima Varzgaliene, Adrienne Heerey, Charlie Cox, Tomas McGuinness, Genevieve McGuire, Jochen WL Cals, Eamonn O'Shea, Maureen Kelly

**Affiliations:** 1 GP Registrar, Western Training Programme in General Practice, Irish College of General Practitioners, Dublin, Republic of Ireland; 2 GP Registrar, Western Training Programme in General Practice, Irish College of General Practitioners, Dublin, Republic of Ireland; 3 GP Registrar, Western Training Programme in General Practice, Irish College of General Practitioners, Dublin, Republic of Ireland; 4 GP Registrar, Western Training Programme in General Practice, Irish College of General Practitioners, Dublin, Republic of Ireland; 5 GP and Programme Director, Western Training Programme in General Practice, Irish College of General Practitioners, Dublin, Republic of Ireland; 6 GP and Assistant Professor, Department of Family Medicine, CAPHRI Care and Public Health Institute, Maastricht University, Maastricht, The Netherlands; 7 GP and Assistant Programme Director, Western Training Programme in General Practice, Irish College of General Practitioners, Dublin, Republic of Ireland; 8 GP and Assistant Programme Director, Western Training Programme in General Practice, Irish College of General Practitioners, Dublin, Republic of Ireland

**Keywords:** Point of care testing (POCT), primary care, general practice, attitudes, benefits, barriers

## Abstract

**Background:**

Studies outside of Ireland have demonstrated that GPs believe point-of-care tests (POCTs) are useful and would like to have more of these tests available in daily practice. This study establishes the views of Irish GPs on this topic for the first time and also explores GPs’ perceptions of barriers to having POCT devices in primary care.

**Aim:**

To establish Irish GPs' perception of the benefits and barriers to POCT use.

**Design & setting:**

A quantitative cross-sectional observational survey of Irish GPs attending continuing medical educational meetings (CME) in November 2015.

**Method:**

Data was collected using an anonymous and confidential questionnaire.

**Results:**

Out of a total of 250, 70% of GPs (*n* = 143) completed the questionnaire. Of these, 92% (*n* = 132) indicated they would like to have access to POCTs. Guidance in decision making 43% (*n* = 61), reduced referral rates 29% (*n* = 42), and diagnosis assistance 13% (*n* = 18) were the main benefits expressed. Cost 45% (*n* = 64) and time 34% (*n* = 48) were the main barriers identified.

**Conclusion:**

This study proved that Irish GPs would also like increased access to POCTs. They feel that these tests would benefit patient care. Unsurprisingly, cost and time were two barriers identified to using POCT devices, which supports outcomes from studies. Radical changes would be required in primary care to facilitate implementation of POCTs and attention must be paid to how the costs of POCTs will be funded. This study may act as a prompt for future international research to further explore this area.

## How this fits in

Studies in Europe have demonstrated that GPs believe POCTs are useful in the primary care setting and would like to have more of these tests available in daily practice. This is the first study to demonstrate that Irish GPs would also like increased access to POCTs. They feel that these tests done quickly in their own practices would assist in making efficient diagnoses, improve management, and reduce referral rates. Perceived barriers to POCT use by Irish GPs are financial cost, time constraints, and quality assurance.

## Introduction

Point-of-care testing has become a hot topic in primary health care.^1^ POCTs are defined as laboratory services using small analytical devices (testing blood, saliva, urine, and faeces) conducted in a patient consultation rather than in a traditional central laboratory.^2^ Therefore, POCTs provide convenient and rapidly available results. Evidence suggest that they facilitate efficient clinical management and reduce patient morbidity and mortality in primary care.^3^ They have the potential to improve efficiencies in many disease areas^4,5–9^ and contribute to cost savings in our overburdened healthcare system.^4,5,10–12^ They also have the potential to enhance patients’ quality of life and increase patient satisfaction with their GPs.^13,14^

If a POCT result is going to influence patient care it must be valid, reliable, and rapid. Use of the results should be evidence-based and cost-effectiveness is essential.^2^ It would be necessary to provide training for GPs on the use and interpretation of these tests and adequate quality control would need to be guaranteed.^15,16^ POCTs, if not properly introduced into primary care in a controlled manner, can lead to inappropriate medical decision making which can adversely affect the patient.^16^

POCT use is not new to Irish primary care. Blood glucose monitoring, urinalysis, international normalised ratios, and urinary beta-human chorionic gonadotropin testing are examples of POCTs that have been successfully incorporated into general practices.^17^ However, there is continuous development of new POCTs and in recent times they have become cheaper, more accurate, and more efficient in producing results.^17,18^ Hence, based on current evidence, the general claim that POCTs are always inferior to standard laboratory test has become a myth.^16^ This progress in POCT technology makes their use in primary care more appealing.

Increased utilisation of POCTs in primary care is likely the way of the future. Studies outside of Ireland have demonstrated that GPs find POCTs useful and would like to have more of them available in daily practice.^15,19^ However, introducing POCTs is likely to have challenges.^16,20,21^ In the recent UK study by Turner *et al,* ^20^ barriers identified included concerns regarding diagnostic accuracy; the impact of testing on clinical skills; costs associated with use and maintenance; and added pressure on clinician time. Responders suggest that quite radical system changes would be required to allow primary care clinicians to capitalise on the potential benefits of POCTs. A systemic review of qualitative studies on attitudes towards point-of-care blood testing^21^ suggested that implementation research evaluating real-life benefits and barriers among countries which have different primary care health system could help in improving the match between clinical needs and technological possibilities. Until this study, there were no data on Irish GP opinion on this topic. A recent large scale national survey of Irish GPs included a detailed description of access to investigations but did not address the topic of POCTs.^22^ This is important, as the Irish funding system is not comparable to international standards and many Irish GP surgeries are more rural than those in more densely populated countries in Europe.^23–25^

There are approximately 2500 GPs practicing in Ireland who deal with over 20 million consultations each year.^26^ Of these, 21% of GPs class themselves as rural practitioners and 42% are classed as urban. The remainder is a mix of both urban and rural.^22^ Without POCTs, there can be a marked delay in getting laboratory results, depending on practice location. Urban practices with daily courier service may have blood results available within the same day. Rural practices may be as far as 80 km from the laboratory and have little or no access to same-day results. The courier service to deliver bloods to the laboratory can be as seldom as twice per week and, once tests are sent, it may take 24–48 hours to get results. As the focus of chronic disease management in Ireland shifts from secondary to primary care, POCTs may offer a convenient and fast alternative to these current delays, especially for the many rural Irish practices. This study investigates these ideas along with GPs’ perceptions of the benefits and barriers to POCT use. Establishing a clinical need or desire for POCT use is a vital step before investing in it. More research is needed to establish if the barriers differ across various healthcare systems. This cross-sectional observational survey aims to establish the level of interest in POCTs; the willingness to invest; and the perceived benefits and barriers of these interesting devices.

## Method

A quantitative cross-sectional observational survey of West of Ireland GPs attending CME meetings in November 2015 was undertaken. A questionnaire was designed (further information available from the authors on request) according to published best practice standards, predominantly using LIKERT scales and Yes/No tick boxes.^27^ A pilot study was conducted prior to study initiation to ensure the questionnaire was concise, user-friendly, and unambiguous.

The CME groups surveyed included rural and urban GPs. A cover letter/participation information form invited the GPs at CMEs to participate and provided participants with study information (further information available from the authors on request). A question regarding informed consent was included in the beginning of the questionnaire (further information available from the authors on request). In adherence with Centers for Disease Control (CDC) guidelines,^28^ questionnaires were voluntary, and all data collected were anonymous and confidential. A period of approximately 10 minutes was allocated at the start of the CME meeting to allow participants to individually complete hard copies of the questionnaire and these were then collected manually by the CME coordinator. This method of data collection was designed in response to the recognised difficulty in getting GPs to complete postal or online research surveys.^24,25,29^ An extensive literature review identified the 10 most requested POCTs in primary care in other countries^19^ and these were listed in the questionnaire as examples of POCTs that may be of use in primary care. Responders were also asked to list any other POCT that they would particularly like to have in their practice. GP demographics, willingness to use POCTs, and the POCTs most sought after were queried. Opinions were sought on perceived benefits and barriers to POCT use in primary care and preparedness to fund POCTs in practice.

### Statistics

Data were collated and analysed using Excel. Statistical analysis was performed using the statistical analysis software program JMP. Descriptive statistics included means, numbers and percentage response rates. Pearson χ^2 ^test was used to compare proportions of responses for categorical data against GP opinions on POCTs. Responses to open-ended and free text comments were grouped together according to topic and analysed qualitatively.

## Results

In total, 205 GPs were expected to attend the CME meetings that were included in this study. Of these, 143 (70%) completed and returned the questionnaire. This represents 5.7% of all practicing Irish GPs. The cohort of this study is somewhat comparable to the whole Irish GP workforce, as the nationwide distribution of urban practices is 42% and the rest are mixed or rural.^22^ In this study, 50% were from rural practices, 37% from urban practices, and the rest were mixed urban/rural practices. Male GPs constituted 53% of the study population (58% nationwide) and 71% belonged to group practices (58% nationwide). Up to 88% of practices had a practice nurse (82% nationwide) and 64% of responders had >10 years of experience working as a GP.^22^ It takes GP practices in the West of Ireland a median of 2.5 days to get a blood result back from a laboratory.

**Figure 1.**
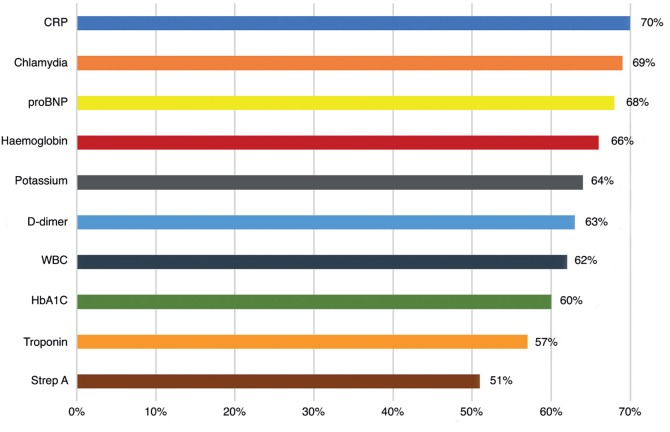
Percentage of GPs who feel that they would use the following POCTs if available in their practice (*n* = 132, 92% of total responders). CRP = C-reactive protein. proBNP = N-terminal prohormone of brain natriuretic peptide. Strep A = Group A streptococcus. WBC = white blood cell.

Of the GPs who completed the questionnaire, 92% (*n* = 132) said they would like to have access to one or more of the POCTs listed (Figure 1). The POCTs most in demand were C-reactive protein ([CRP], 70%), chlamydia (69%) and N-terminal prohormone of brain natriuretic peptide ([proBNP], 68%). Most GPs felt that they would use POCTs regularly, with only 1% declaring that they would never use them.

Guidance in decision making 43% (*n* = 61) was the main benefit expressed. The main barrier to using POCT devices identified by participants was cost 45% (*n* = 64). (Table 1).

**Table 1.** Main benefits for and barriers to GPs using POCTs in their practice

Benefits	Responders, *n* (%)	Barriers	Responders, *n (%*)
Guidance in decision making	61 (43)	Cost	64 (45)
Reducing referral rates	42 (29)	Time	48 (34)
Aid diagnosis	18 (13)	Quality assurance	13 (30)
Increased patient satisfaction	13 (9)	Increased patient expectations	6 (8)

Nearly half of GPs (48%, *n* = 68) were prepared to spend money on POCT equipment. A median of €500 (interquartile range [IQR] €200–€500) was what GPs were willing to spend on a POCT device. An equal number of GPs indicated that they would not be willing to spend any money on POCT equipment, and that they would only use POCTs if the Irish Health Service Executive (HSE) or another governing body funded the equipment for them. The remaining 5% of responders were unsure if they would spend any money on these devices. Only 28% of GPs said that they would be willing to charge patients for use of a POCT, with a median charge of €20 (IQR €10–€25). GPs with <10 years of experience were significantly more likely to invest in POCTs (Pearson χ^2^ = 10.1 [*P* = 0.0125]). There was no other significant difference between willingness to invest in POCT devices and any other practice demographics (including sex, type of practice [single versus group; rural versus urban], distance from emergency department and availability of a practice nurse).

## Discussion

### Summary

This is the first study to assess Irish GPs' attitudes to POCT use in primary care. The majority of GPs (92%) from both rural and urban practices would like to have access to more POCTs and think they would benefit patient care. Guidance in decision making and reducing referral rates were the main benefits expressed. Unsurprisingly, cost and time were two barriers identified to using POCT devices.^16,20,21^ Most GPs would only use them if an external body, such as the Irish HSE, funded the equipment. Ireland has both a comprehensive, government-funded, public healthcare system and private healthcare system, depending on patient income. There is no way for GPs to get reimbursement from the government for the cost of POCTs at present. Therefore, in the context of reductions in health service funding, and the importance of primary care commissioning, attention must be paid to how the costs of POCTs will be funded.

### Strengths and limitations

This the first study to assess Irish GPs' attitudes to POCT use in primary care. Moreover, it gives GPs' views from a different primary care health system as suggested by Jones *et al*’s 2013 systematic review, which included only high income country settings.^21^ Furthermore, it explores real-life barriers among GPs and supports outcomes from a recent UK study by Turner *et al* ^20^ which suggested that radical changes would be required to enable clinicians to take advantage of the potential benefits of POCTs in primary care. Also, attention must be paid to how the costs of POCTs will be funded. It may not be possible to generalise the results of this study to GPs internationally, due to variability in funding structures and current access to laboratory services. However, it is very likely that most worldwide GPs would share these concerns. This study may act as a prompt for future international research to further explore this area.

A study limitation was that only GPs attending CME groups in the West of Ireland were included. This facilitated a good response rate (70%), but may have introduced bias. CME groups were targeted as it was felt that a higher response rate would be achieved than by using traditional ways of obtaining data, such as posting questionnaires manually or sending them electronically. It has been demonstrated that questionnaire distribution via CME can yield a response rate as high as 97%.^29^ Two other GP projects have been successfully completed using questionnaires distributed at CME meetings.^24,25^ GPs motivated to attend CME groups may be more open to new practices, such as the increased use of POCTs, compared to those who do not make the time to attend these meetings. However, this study's cohort representativeness (practices location, practice set, GPs' sex and age) is somewhat comparable to the whole Irish GP workforce as nationwide,^22^ which should reduce study bias.

### Comparison with existing literature

Increased utilisation of POCTs is likely to play a major role in the future of primary care worldwide. Spano *et al* ^19^ demonstrated that GPs find POCTs useful and would like to have more available in daily practice. However, there have been very few attempts to determine why GPs hold these beliefs and what GPs' perception of barriers to having POCT devices in primary care are.^20,21^ To the authors' knowledge, willingness to spend any money on POCT equipment has also not been studied. Nor was there previous research on whether or not Irish GPs desire increased access to POCTs. More research was needed to establish if the barriers differ across various healthcare systems. This study established that Irish GPs want the same access to POCTs as their overseas colleagues.^19,20^ GPs preferably want to decide on management within the 10-minute consultation for acute conditions, as previously discussed in the *BJGP*.^30^ Their most desired POCTs were CRP, chlamydia, and proBNP, which was similar to that of their European colleagues. The perceived barriers to POCT institution in primary care include the financial cost and time taken to conduct POCTs.

Literature suggests POCTs can facilitate efficient clinical management,^3,20,21^ increase patient satisfaction with their GPs,^14^ and reduce referral rates. This research demonstrates that Irish GPs also hold this view. In practice, there is evidence that this is the case for POCTs used in conjunction with guidelines for their indication and implementation, specifically CRP and D-Dimer,^5,31^ but evidence is lacking for other POCTs as yet. In fact, one study demonstrates that the use of POCTs without guidelines may increase adverse outcomes.^2,15^

### Implications for research and practice

For POCTs to become a reality in primary care, their benefit must outweigh their potential risks. Results must be accurate, reliable, rapidly available, and cost-effective.^2^ Existing literature suggests that POCTs are most beneficial and safe when guidelines are implemented to guide indication and interpretation. Further research generating evidence-based guidelines to optimise the clinical role of POCTs and ensure patient safety would be required. Considerable barriers to uptake prevail at the clinician, patient, and system levels need be considered.^20^ In addition, cost-effectiveness analysis would need to be performed to help determine if POCTs are a worthwhile investment. Funding models need to be generated in order for POCTs to become commonplace in Irish primary care. Negotiations between GPs and the HSE may be the route to instituting POCTs.

In conclusion, increased utilisation of POCTs in primary health care is likely to play a significant role in the future treatment of patients. This study established that the majority of participating GPs, though aware of the need for care in their implementation and certain barriers to their use, are interested in principle in adopting POCTs into their practice.
